# A human-based assisted reproduction protocol for the menstruating spiny mouse, *Acomys cahirinus*

**DOI:** 10.1371/journal.pone.0244411

**Published:** 2020-12-28

**Authors:** Jarrod McKenna, Nadia Bellofiore, Sally Catt, Mulyoto Pangestu, Peter Temple-Smith

**Affiliations:** 1 Education Program in Reproduction & Development, Department of Obstetrics & Gynaecology, School of Clinical Sciences, Monash University, Clayton, Victoria, Australia; 2 The Ritchie Centre, Hudson Institute of Medical Research, Clayton, Victoria, Australia; Infertility Unit, ASST Lariana, ITALY

## Abstract

The Egyptian or Common spiny mouse (*A*. *cahirinus*) is the first rodent species to show human-like menstruation and spontaneous decidualisation. We consider from these, and its other, human-like characteristics that this species will be a more useful and appropriate small animal model for human reproductive studies. Based on this, there is a need to develop specific laboratory-based assisted reproduction protocols including superovulation, in-vitro fertilisation, embryo cryopreservation and transfer to expand and make this model more relevant. Because standard rodent superovulation has not been successful in the spiny mouse, we have selected to test a human protocol. Female spiny mice will receive a subcutaneous GnRH agonist implant and be allowed to recover. Menstrual cycle lengths will then be allowed to stabilize prior to ovarian stimulation. After recovery, females will be injected IP once a day for 4 days with a FSH analogue, to induce follicular growth, and on day 5 will be injected IP with a hCG analogue to trigger ovulation. Females will either be culled 36hrs after trigger to collect oocytes or immediately paired with a stud male and two cell embryos collected 48hrs later. Mature oocytes will be inseminated using fresh spiny mouse spermatozoa and all in-vitro grown and in-vivo collected two cell embryos will be cryopreserved using methods developed in a close spiny mouse relative, the Mongolian gerbil. For embryo transfer, vitrified embryos will be rapidly warmed and non-surgically transferred to surrogate mice. Surrogates will be monitored until pregnancy is apparent (roughly 30 days) and then left undisturbed until birth, 38–40 days after transfer. By successfully developing robust assisted reproduction protocols in *A*. *cahirinus* we will be able to use this rodent as a more effective model for human reproduction.

## Introduction

The Egyptian or common spiny mouse (*A*. *cahirinus)* is a small, desert-dwelling rodent native to northern Africa and the Middle East. Spiny mice exhibit several unique, sometimes human-like, characteristics among rodents, including a relatively long gestation (39 days; [1]), small litter sizes (1–4; observations from our colony) and the recent discovery of human-like menstruation [2]. This latter trait in particular, is expressed in <2% of all mammalian species and is primarily found in higher-order primates such as gorillas and orang-utans [3]. Although other non-primate species including the elephant shrew [4] and short-tailed fruit bat [5] exhibit menstrual-like events, *A*. *cahirinus* is, to our knowledge, the only species with human-like, cyclical menstruation. However, as the spiny mouse is a comparatively novel laboratory species, limited information is available regarding their reproductive physiology and endocrinology and how this compares to other menstrual species. Despite this, human-like, cyclical menstruation in spiny mice has established this species as an important new small animal model for studies into human reproductive and gynaecological disorders.

While other aspects of reproduction, such as the later stages of gestation, in *A*. *cahirinus* have been well documented [6–9], protocols for various assisted reproductive techniques (ARTs), including gamete and embryo cryopreservation, in-vitro fertilisation (IVF) and embryo transfers (ET) have yet to be developed in this species. Access to robust ARTs in *A*. *cahirinus* will allow for more efficient in-vitro and in-vivo research to understand better embryogenesis, implantation and pregnancy to evaluate further its role as a small animal model of human reproduction.

In women, superovulation involves two primary steps, 1) suppression of ovarian function by gonadotrophin-releasing hormone (GnRH) agonist or antagonists followed by, 2) direct stimulation using exogenous follicle stimulate hormone (FSH) [10, 11]; a feature absent from the original *Acomys* protocol [12]. Ovarian downregulation using Deslorelin, a potent GnRH agonist, has been successful in several mammalian species [13–17] and we hypothesise that Deslorelin will also be effective for producing large numbers of mature metaphase II oocytes in female spiny mice from our colony.

Successful ovulation and IVF of mature oocytes will then allow us to produce pre-implantation embryos that can either be frozen or transferred fresh to surrogates. In light of advances in human embryo vitrification, frozen-embryo transfers (FET) are now the preferred technique in human IVF [18]. Subsequent development of embryo vitrification in this *Acomys* species will streamline embryo transfer procedures, and provide the foundation for future creation of transgenic strains and for improved breeding efficiencies for captive spiny mouse colonies.

Our aim with this protocol is to develop a successful agonist-based superovulation technique for *A*. *cahirinus* that will provide large numbers of oocytes for IVF, and also embryos for culture, cryopreservation and transfer, and enable us to more effectively use the spiny mouse as a model for human reproduction.

## Methodology

### Animals

Monash University/Monash Medical Centre Animal Ethics Committee have approved the experimental use of this protocol (MMCB/2019/10). The animals for this study are sourced from our research colony of spiny mice maintained under a 12:12 hour light:dark cycle at 25–27°C and humidity of 30–40% [19]. Sexually mature mice (>60 days old) are housed in groups up to nine per cage (female only) or as breeding pairs. Wood shavings are used to line cage floors and plastic tunnels, rings and tissue paper provide environmental enrichment. Food (rat and mouse cubes; Specialty Feeds, Glen Forest WA) and water is provided freely at all times, with weekly supplements of fresh carrots and celery.

### Sample size and experimental design

Female spiny mice on average deliver litter sizes of 3 pups (our colony observations) but can range from 1–5 naturally. We have based our sample size calculations (80% power, alpha 0.05, sigma 2.5) on collecting 3 eggs per female from controls and 6 eggs from stimulated animals. Thus, the required number of females to show significant increases in egg/embryo numbers compared to controls is 9. This also includes an additional 3 animals to account for natural biological variation.

18 males are required for each of the 18 females which will be paired to collect embryos.

The success of the NSET is approximately 30% in laboratory mice [20]. To achieve 3 sets of live births, and show a significant effect of treatment, we will need a minimum of 12 surrogate spiny mice per group.

**Fig 1:**
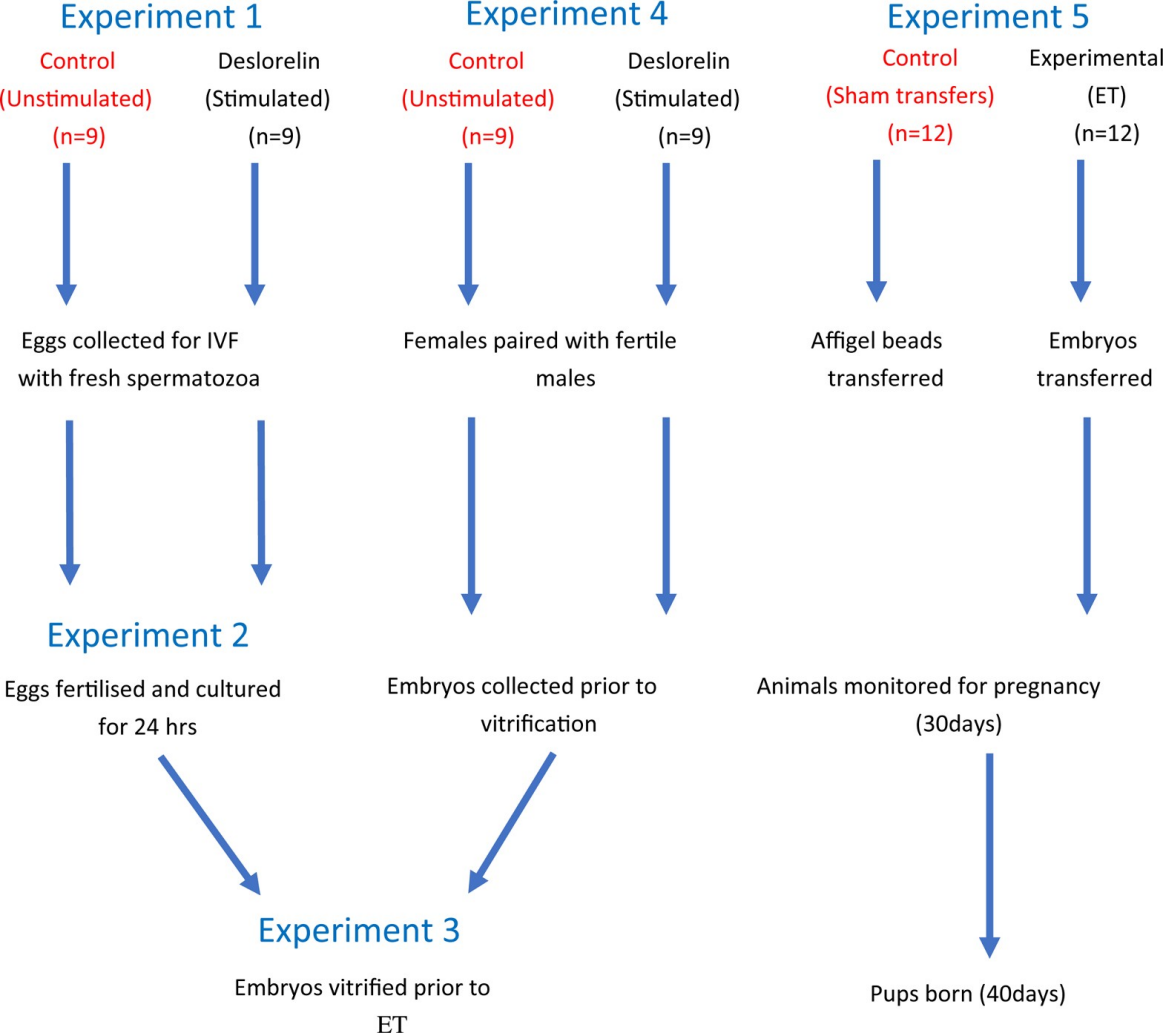
Overall experimental design. Experiment 1) Oocytes will be collected from stimulated (experimental) and unstimulated (control) animals prior to IVF. Experiment 2) Oocytes will be fertilised with fresh spiny mouse spermatozoa and cultured to the two cell stage prior to vitrification. Experiment 3) Embryos will be vitrified, stored and then thawed prior to ET. Experiment 4) Embryos will be collected from stimulated (experimental) and unstimulated (control) animals prior to vitrification. Experiment 5) Embryos will be thawed and transferred to surrogate female spiny mice. Dams will be monitored for pregnancy (~30 days) and for live births (~40days). A group of sham-transfer controls have affigel beads transferred in place of embryos.

The experimental design consists of five experiments (Fig 1) conducted in three phases (Table 1):

1. to develop a novel agonist-based superovulation protocol for the reliable collection of spiny mouse oocytes.
2. to define an IVF protocol for mature spiny mouse oocytes using fresh or cryopreserved epididymal spermatozoa.
3. collect viable two cell embryos from superovulated, mated spiny mice.
4. to successfully vitrify and warm *in vivo* flushed and in-vitro grown two cell embryos.
5. to compare the pregnancy success rates and birth outcomes of in-vitro grown and in-vivo collected embryos after non-surgical embryo transfer (NSET).

**Table 1:** Research timeline for ART development in *Acomys cahirinus*.

		Months								
		1	2	3	4	5	6	7	8	9
Phase 1	Transfer training									
	Implant surgery training									
	SHAM transfers									
Phase 2	Oocyte donor control collections									
	Surrogate transfers + litter down									
	Embryo donor control collections									
	Surrogate transfers + litter down									
Phase 3	Oocyte donor implant surgery									
	Embryo donor implant surgery									
	Oocyte collections									
	Surrogate staging + litter down									
	Embryo collections									

Experiments are conducted in three phases: Phase 1 consists of technique training and embryo transfer controls, phase 2 includes control oocyte and embryo collections, whilst phase 3 includes experimental oocyte and embryo collections.

### Tissue collections

Mature male mice are euthanised by isoflurane inhalation and the epididymides are removed through a midline abdominal incision by retracting each epididymis through its inguinal canal into the abdominal cavity. Each cauda epididymis is isolated, incised several times with fine iridectomy scissors and placed in warmed collection tubes containing 500uL of pre-equilibrated MT6 fertilisation medium (37°C; 5% O_2_, 6% CO_2_, 79% N_2_; modified Tyrode’s solution [21]). Spermatozoa are allowed to swim out and begin capacitation in MT6 in a humidified incubator (37°C, 5% O_2_, 6% CO_2_) for at least 30 minutes to provide a sperm sample with rapid forward motility for IVF (Experiment 3).

Oocytes are collected from females euthanised by isoflurane inhalation; their oviducts are removed immediately post-mortem and placed into 2mL of warmed (37°C) HEPES-based handling medium (M2; Sigma, CAT #M7167). The oviducts are then placed into a 60mm culture dish (Sigma) on a heated stage at 37°C for dissection. Each oviduct is gently opened using sharp micro-forceps to release cumulus oocyte complexes (COCs). The COCs are placed into a 50uL drop of pre-equilibrated MT6 and transferred into an incubator (37°C, 5% O_2_, 6% CO_2_) prior to IVF (see Experiment 3). For embryo collections, female spiny mice are killed and dissected, as described for oocyte collections above. Embryos are released into a 60mm culture dish (Sigma, CAT #SIAL0166) on a heated stage containing warmed M2 handling medium (37°C) and washed three times in fresh 50uL drops prior to vitrification (see Experiment 3).

### Experiment 1: Unstimulated control oocyte collections

**Fig 2:**
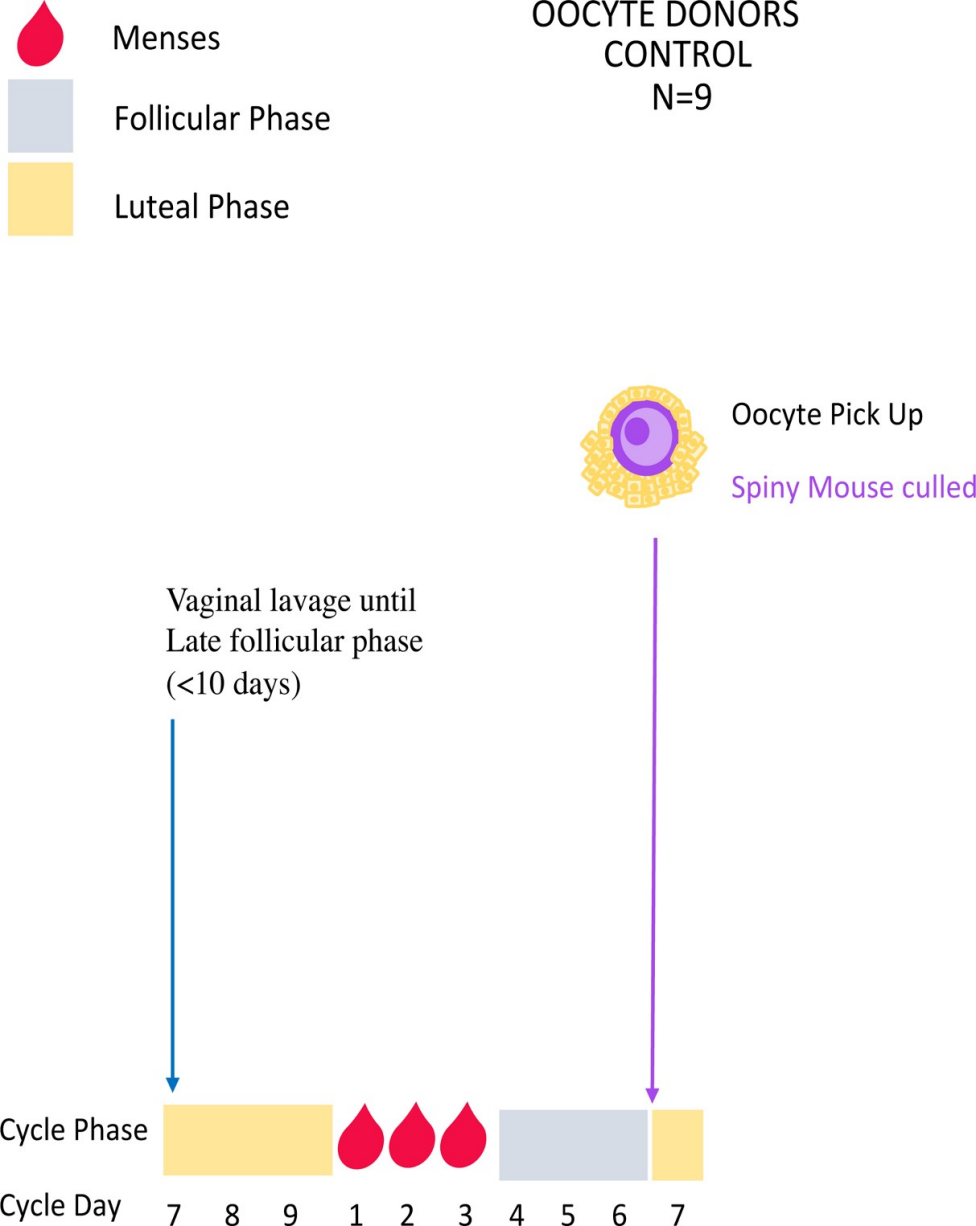
Control unstimulated oocyte collections. Mice are lavaged daily until the late follicular phase (~day 6 of the menstrual cycle) when they will be killed and oocytes collected for use in IVF (Experiment 2).

Spiny mouse menstrual cycles are monitored and characterised using vaginal cytology to identify 1) cycling individuals and 2) those in late follicular phase according to published criteria [2, 22] prior to hormone treatment. Females are then killed when they reach the late follicular stage, and oocytes collected for use in IVF, embryo vitrification and ET (Figs 1 and 2; Experiments 2–3, 5).

### Experiment 1: Deslorelin stimulated oocyte collections

The vagina of females undergoing superovulation is lavaged as described previously, and the vaginal cytology used to identify menstruation (day 1–3 of the cycle). A subcutaneous Deslorelin implant (Virbac, Australia) is then surgically inserted into each menstruating female spiny mice as follows:

1. Each female receives 5mg/kg subcutaneous Carprofen analgesia immediately prior to surgery. Anaesthesia is induced with 5% isoflurane in room air via an automated anaesthetic machine (Univenter 400 anaesthetic unit; Zejtun, Malta). When adequately anaesthetised, the level of isoflurane is reduced to ~2.0–2.5% to maintain an appropriate level of anaesthesia during surgery. Each female is placed in a supine position on a warming pad (37°C) and depth of anaesthesia monitored using foot and eye reflexes.
2. A 3mm interscapular incision is made in the skin and a 2cm x 2cm pocket formed with blunt dissection. The incision is flushed with 0.2mL topical analgesic (Bupivacaine) and interrupted the Deslorelin implant inserted into the pocket which is closed with 6/0 Ethilon sutures (Ethicon, Australia).
3. The nails on the front paws of the anaesthetised females will be trimmed to prevent scratching causing wound and skin tears and removing sutures.
4. Females are monitored every 2hrs for 8hrs after surgery, twice daily thereafter for 3 days and daily until 7 days after surgery.
5. Implanted females are allowed 27 days to recover before commencing gonadotrophin injections.

**Fig 3:**
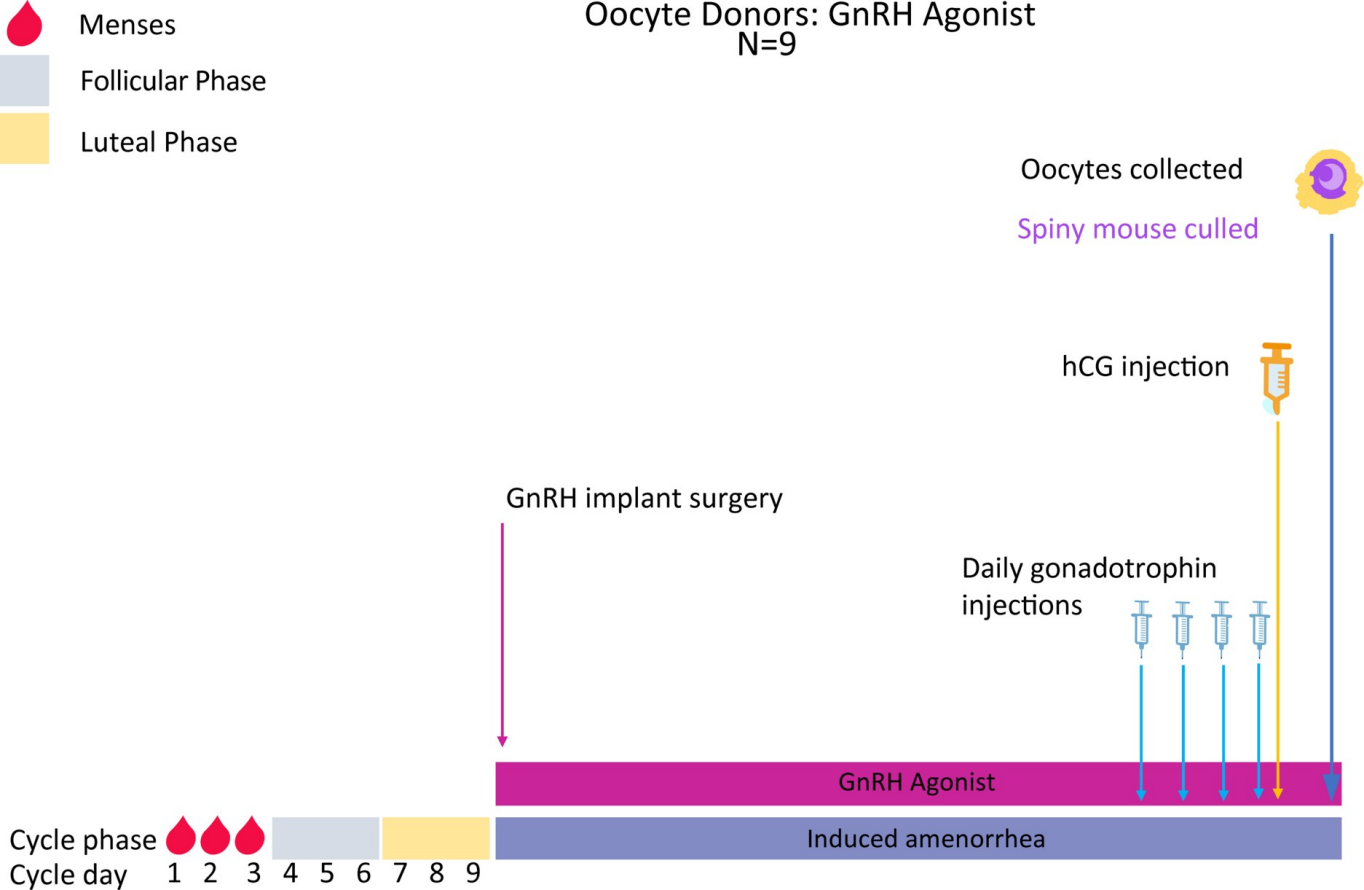
Superovulation protocol and oocyte collections in Deslorelin implanted mice. Mice are vaginally lavaged until the menstrual phase (~ day 1–3 of the cycle) and a Deslorelin implant then subcutaneously implanted into each mouse. All mice will be given 3 cycle lengths (27 days total) to recover from surgery and for the agonist implant to have taken effect. Mice will then be injected IP once daily for 4 days with Gonal-F followed by a single injection of Ovidrel on day 5. Females are culled 36 hours later and oocytes collected for use in IVF.

**Fig 4:**
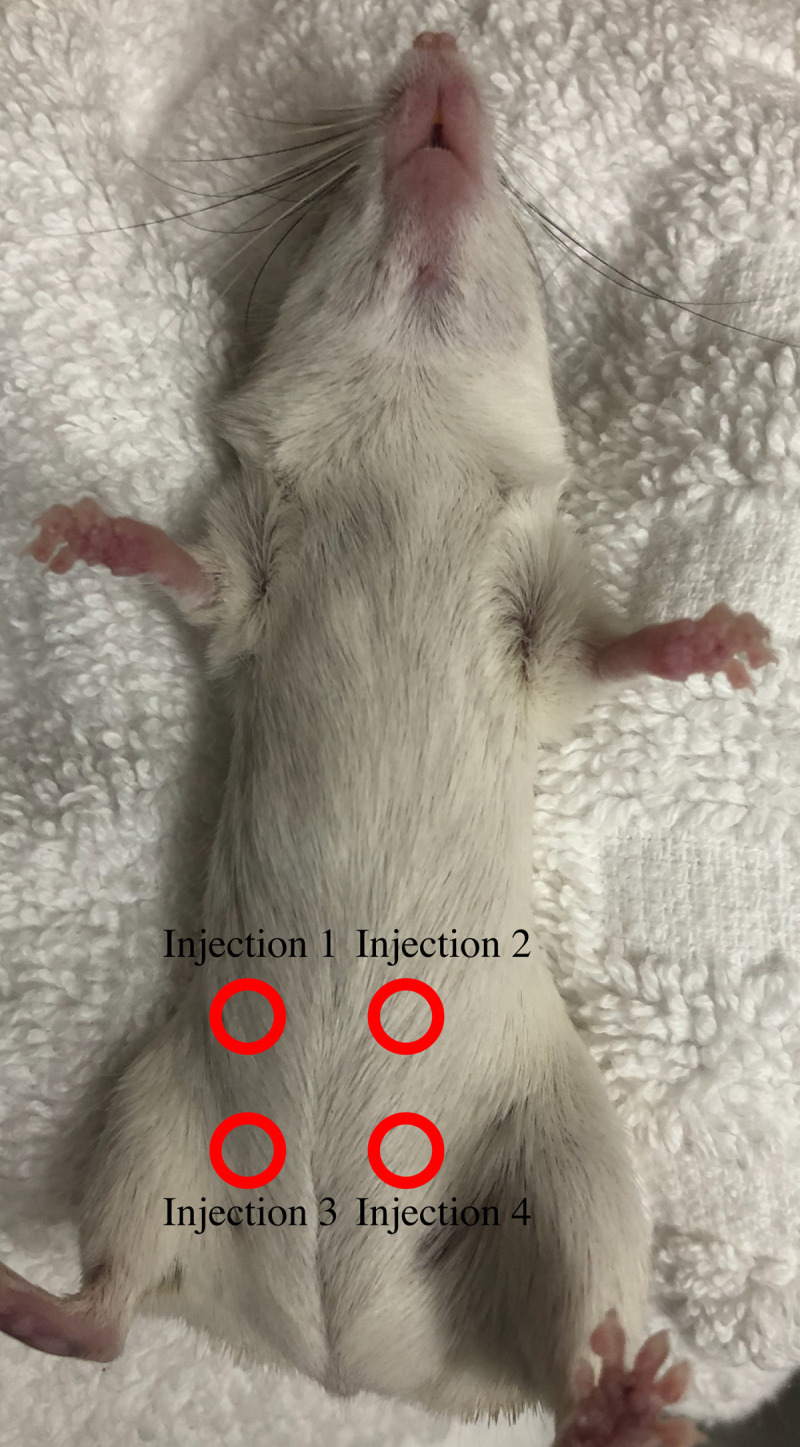
Rotation of injection sites for hormone IP injections. Injection sites are rotated across the lower abdomen of spiny mice to prevent injury from repeated injection sites.

After recovery, females are injected (intraperitoneal; IP) once a day for 4 days with Gonal-F (Merck Serono, Australia), an FSH analogue, to induce follicular growth (Figs 3 and 4). On Day 5 of treatment, females are injected IP with Ovidrel (Merck Serono, Australia) to trigger ovulation. Females are killed 36hrs later by isoflurane inhalation, the ovaries and oviducts removed by fine dissection and ovulated oocytes released from the oviducts into fertilisation medium (MT6) for use in IVF and, subsequently, embryo vitrification and ET (Figs 1 and 2; Experiments 2–3, 5).

### Experiment 2: In-vitro fertilisation (IVF)

A 10uL aliquot of highly motile spermatozoa is aspirated, after a 30-minute swim-up at 37°C from the top of the collection tube for motility assessment as previously reported [23]. A volume (roughly 2-4uL) containing at least 250,000 progressively motile spermatozoa is added to the drop of MT6 containing the COCs and returned to the incubator. After three hours incubation, the inseminated oocytes are washed 3 times in 50uL drops of M16 culture media (Sigma, CAT #M7292) and cultured in fresh media droplets for 24hrs in a humidified incubator (37°C, 6% CO_2_, 5% O_2_). Twenty-four hours after insemination, fertilisation rates are estimated by the number of two cell embryos produced divided by the number of oocytes inseminated. Embryos are removed from M16 and washed 3 times in handling media (M2) prior to vitrification (Experiment 3).

### Experiment 3: Embryo vitrification

In-vitro grown and in-vivo collected two cell embryos are vitrified and stored to better manage the timing of embryo transfers with IVF and embryo collections. Embryo vitrification will be performed according to the methods developed by Mochida, Wakayama, et al. [24] for Mongolian gerbil embryos with minor modifications. Up to 20 embryos are placed in 500uL of equilibration solution [ES; M2 containing 20% v/v ethylene glycol (EG), 30% Ficoll w/v and 0.5M sucrose) for two minutes at room temperature. Embryos are then transferred to 500uL of vitrification solution [ES; M2 containing 40% v/v ethylene glycol (EG), 30% Ficoll w/v and 0.5M sucrose) for thirty seconds at room temperature. The embryos are then aspirated inside 0.25mL cryopreservation straws (INV technologies, India) and a 1cm air gap aspirated immediately after the VS. The straws are then sealed with polyvinyl alcohol (PVA) and immersed directly in liquid nitrogen (LN2) for storage.

For warming, each straw is removed from LN2 and placed in a 22°C waterbath for 10 seconds. The straws are then cut between the PVA seal and the VS, and embryos expelled into 800uL of M2 medium containing 0.5M sucrose. The embryos are then transferred to a fresh drop (50uL, 5embryos/drop) of M2 medium containing 0.5M sucrose for five minutes. After warming, the embryos are transferred to fresh 50uL drops of M2 medium prior to ET.

### Experiment 4: Embryo collections from unstimulated control and Deslorelin-treated females

**Fig 5:**
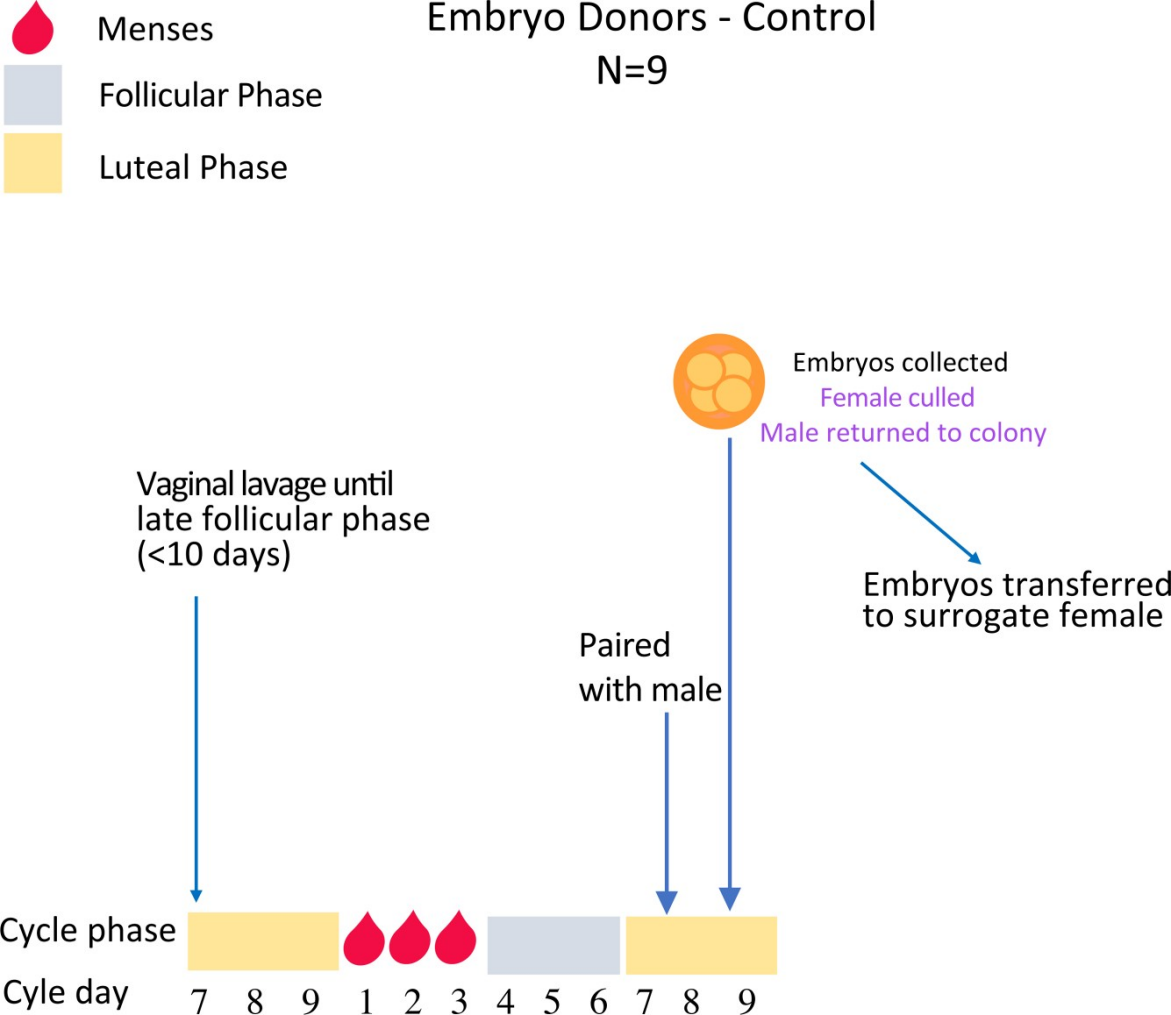
Embryo collections from unstimulated spiny mice. Females are lavaged daily until the late follicular phase (~day 6 of the cycle) when they will be paired with a fertile male and left undisturbed to mate overnight. Female mice are then killed 48hrs later and embryos collected for immediate vitrification (Experiment 3).

After vaginal lavage, when females reach the late follicular phase, they are paired in the morning (Day 6) with a sexually mature stud male with proven fertility and left undisturbed to mate. Females are killed 48hrs later and embryos collected for immediate vitrification (Figs 1 and 5).

**Fig 6:**
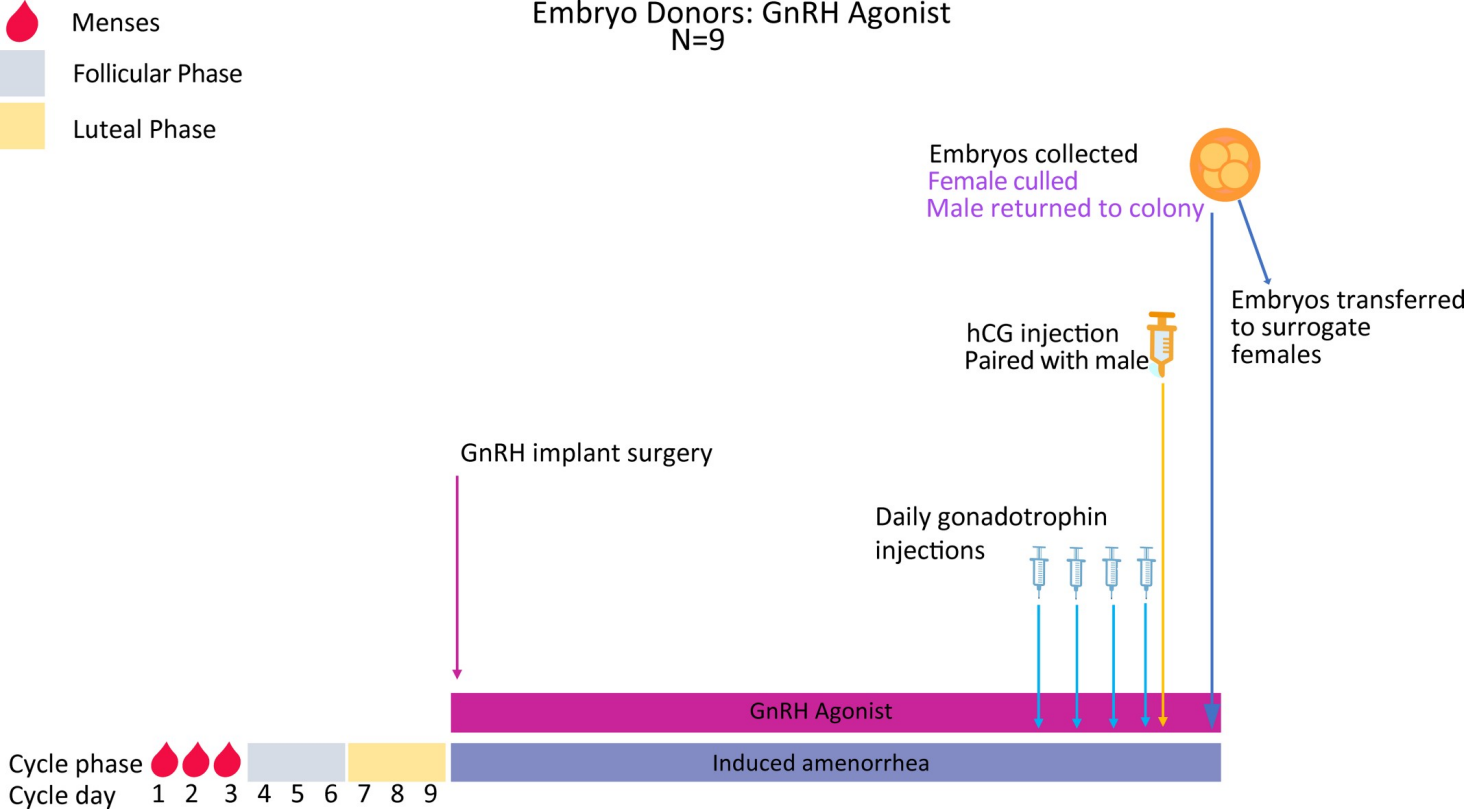
Superovulation protocol and embryo collections in Deslorelin implanted spiny mice. Females are vaginally lavaged until the menstrual phase (~ day 1–3 of the menstrual cycle) and a Deslorelin implant then subcutaneously implanted into each mouse. Females will be allowed 3 cycle lengths (27 days total) to recover and for the agonist implant to have taken effect. They will then be injected IP once daily for 4 days with Gonal-F followed by a single injection of Ovidrel on day 5. Females are then paired with a fertile male and left undisturbed to mate overnight. Forty-eight hours after the Ovidrel injection, the females are killed, embryos collected and immediately vitrified (Experiment 3).

As per oocyte collections above, the female spiny mice in this protocol receive Deslorelin slow-release implants. After recovery from implant surgery (3 cycle lengths; Fig 6), each female is placed on the same routine of 4 daily injections of gonadotrophins followed by an ovulation trigger. Immediately after the ovulation trigger, each female is paired with a male, and embryos are collected 48hrs later for vitrification (Experiment 3).

### Experiment 5: Embryo transfer

Non-fragmented, morphologically normal two cell embryos are selected and transferred using a NSET device (Paratechs, USA) as previously reported [20] but with the following modifications. As spiny mice are unable to become pseudopregnant [25], surrogates are lavaged daily for up to 7 days to identify the mid-late luteal phase [2] at which females are most receptive to implantation and pregnancy. A maximum of 4 embryos, as opposed to the 20 transferred in a previous study [20], are transferred into surrogate spiny mice. Spiny mice have comparatively small litter sizes (average 2–3; [1, 19]) compared to laboratory mice (average 8–9; [26]), and thus, transfer of more than four embryos may negatively affect pregnancy or birth outcomes.

**Fig 7:**
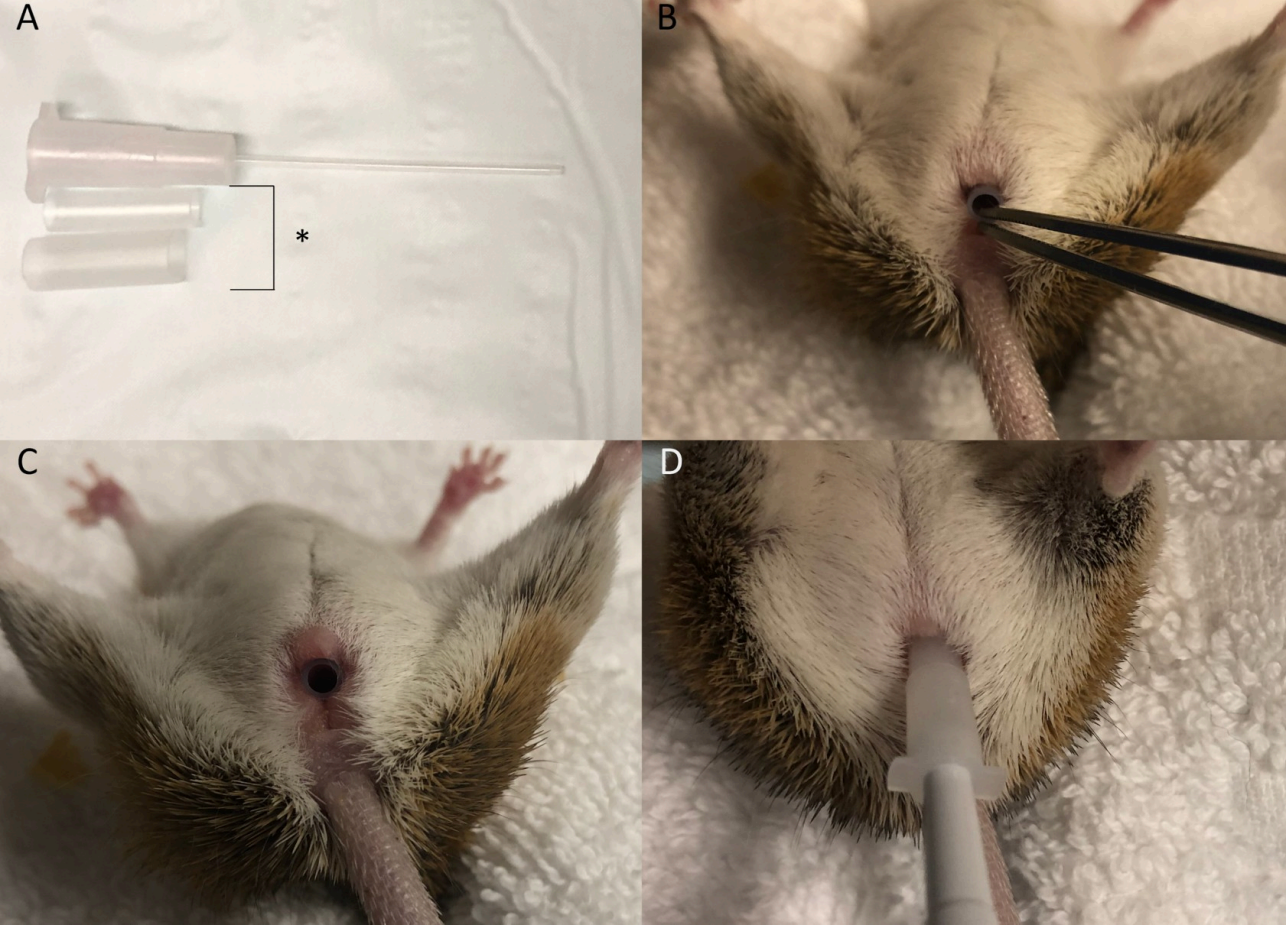
NSET transfer device and handling procedure. A) Both speculum (*) and the NSET cannula, B) Insertion of the smaller speculum, C) Speculum fully inserted in the vaginal canal, D) Insertion of NSET needle through the speculum and cervical opening.

The NSET is attached to a 2.5uL pipette (Eppendorf) and set to 1.0uL. The plunger is depressed to half the first stop, embryos aspirated, and the pipette reset to 1.2uL to allow a bubble to form at the tip of the NSET. Spiny mice are held supine and the speculum inserted into the vagina to open the canal prior to embryo transfer (Fig 7A–7C). If needed, the smaller speculum is replaced by the larger speculum to better visualise the cervix. The NSET cannula is inserted into the vagina and passed through the cervix until the base of the cannula reaches the base of the speculum (Fig 7D). The pipette plunger is depressed to the first stop to expel the embryos, and the device slowly withdrawn as the plunger is depressed to the second stop to prevent embryos being aspirated back through the cervix.

**Fig 8:**
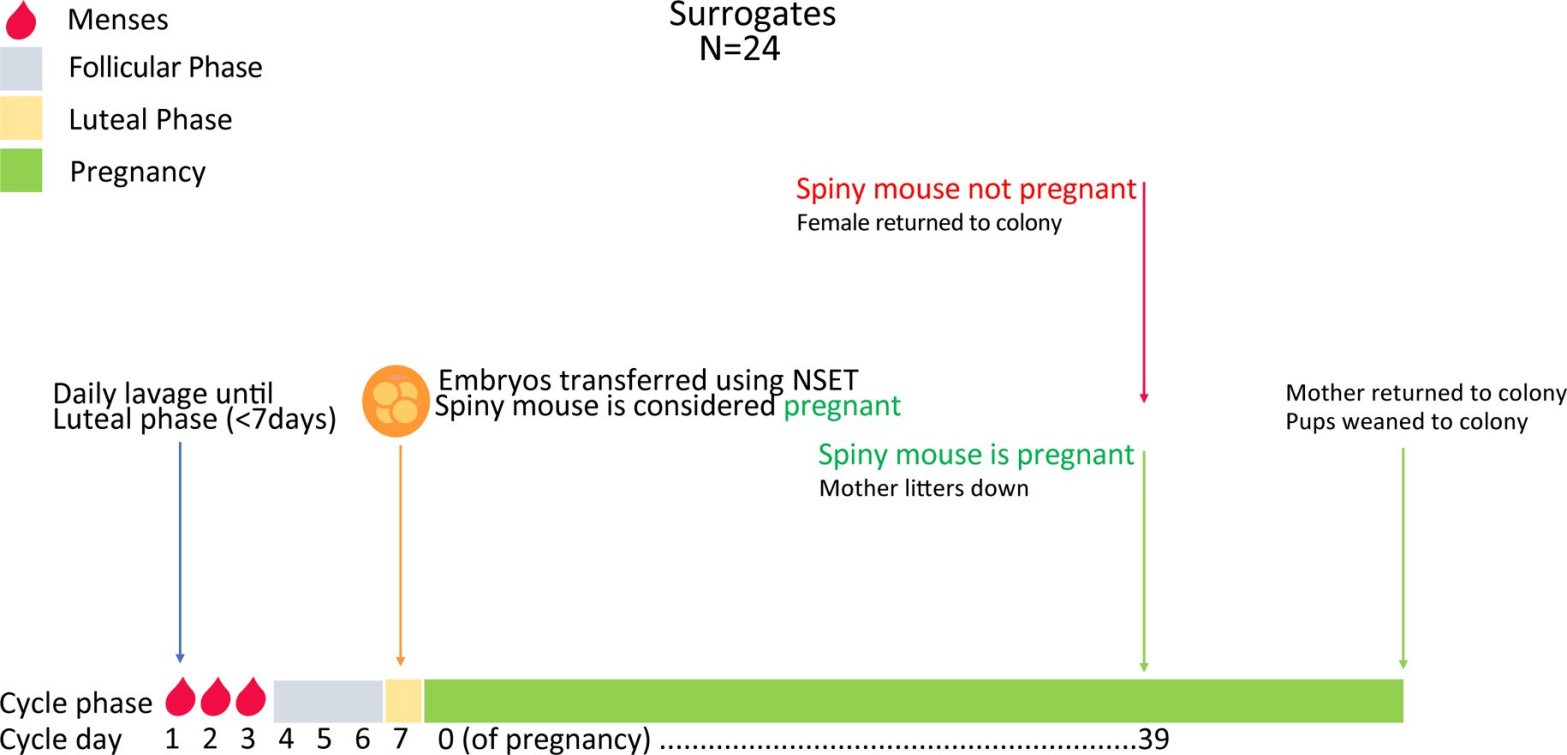
Embryo transfer procedure for surrogate spiny mice. Mice are vaginally lavaged until the luteal phase (~ day 7 of the menstrual cycle) and a maximum of 4 embryos will be transferred per female using the NSET device. Surrogate mice are presumed pregnant and will be monitored for external signs of pregnancy (~30 days). If mice are considered non-pregnant, they are removed from experimentation and returned to colony. If mice are pregnant, they are left to litter-down (~8–10 days later).

The NSET tip is then checked under a dissecting microscope to confirm that all embryos have been transferred into the uterus. Each female is then returned to her respective cage and monitored twice daily for 3 days from the day of transfer and then daily until pregnancy is obvious (~30 days gestation; observations from our colony). The females are then left undisturbed until birth (roughly 38–40 days; Fig 8) or returned to colony if non-pregnant.

In order to observe any effects of the NSET device and transfer technique on spiny mouse behaviour and gestation, a control group of sham-treated animals will have non-toxic artificial blue beads (Affi-gel) transferred using the NSET in place of embryos.

### Statistical analysis

1. All data will be tested for normality using the Shapiro-Wilk normality test prior to further analysis.
2. Number of oocytes and embryos collected from control and experimental animals will be compared statistically using a one-way ANOVA and the Tukey test used for post-hoc analysis; significance is set at p<0.05
3. IVF and developmental rates following unstimulated and stimulated oocyte collections will be compared using a one-way ANOVA and the Tukey test used for post-hoc analysis and significance set at p<0.05.
4. Embryo survival rates post-warming will be compared between i*n-vitro* grown and in-vivo collected groups using an unpaired t test and significance set at p<0.05.
5. Live birth rates after embryo transfer will be compared between 1) control (unstimulated) and stimulated cycles; 2) In-vitro produced and in-vivo collected embryos. All live birth data will be analysed using a two-way ANOVA and the Tukey test used as post-hoc analysis with significance set at p<0.05

## Discussion

Collection of progressively motile spermatozoa, and sufficient numbers of oocytes and embryos are critical steps for developing assisted reproductive techniques in any species. Currently, the most successful murine superovulation protocol involves single injections of equine chorionic gonadotrophin (eCG), to stimulate follicular growth, and human chorionic gonadotrophin (hCG) for ovulation induction [27]. However, the efficacy of this protocol is known to vary with age, genetic background and stage of oestrus cycle [28–31]. Although *A*. *cahirinus* is a rodent, no other rodent species, including other *Acomys* species, have been shown to menstruate. We consider that failure of our previous attempts to superovulate this species using the published A. *cahirinus* superovulation protocol [12] is due to its unusual reproductive strategy and hormonal profile [32]. Here, because of many similarities between spiny mouse and human reproduction, we have developed a novel superovulation and embryo transfer protocol for *A*. *cahirinus* based on a human agonist cycle.

Although GnRH agonists have been used in superovulation protocols for other menstruating species [33–36] such as baboons [35] and cynomolgus monkeys [33–36] they were injected, not implanted, with the GnRH agonists. In our experience, simultaneous hormone and agonist injections are likely to cause excessive, investigator-induced handling stress [37] and negatively affect superovulation outcomes. Given the human-like menstruation in *A*. *cahirinus*, its small size and high susceptibility to stress, an agonist-based superovulation protocol using a GnRH agonist implant is considered a more appropriate and potentially successful approach to superovulation in this species.

Embryo vitrification is an essential murine ART for preserving strain genetics, colony management and streamlining ET procedures [38]. Although murine ETs are often performed using in-vitro grown blastocysts [20, 39], Momozawa, Matsuzawa, et al. [40] showed that healthy offspring can be produced either from vitrified blastocysts or two cell embryos. Interestingly, *A*. *cahirinus* experience an in-vitro developmental block at the four cell stage (unpublished; observations from our lab). Currently, the developmental capacity of blocked four cell embryos and the in-vitro requirements to progress past this stage are unknown. Thus, in-vitro grown, or in vivo collected, two cell embryos will be immediately vitrified prior to ET using a published vitrification method for two cell embryos from Mongolian gerbils; the closest relative of A. cahirinus [41–43].

Embryo transfers in mice, unlike in humans, are more commonly performed under general anaesthesia and usually embryos are surgically transferred directly into the oviduct [44–46]. Although NSET was first described nearly 70 years ago [47], poor live birth rates (8.5%) hindered its adoption compared to surgical embryo transfer (15–35%; [48]). Recently, however, NSET has become more successful with studies reporting comparable pregnancy and live birth rates to surgical methods [20, 39]. Thick, rigid tubing used by Beatty [47] were replaced with more flexible plastic tubing to more easily pass through the cervix and to prevent side-effects like uterine scratching and puncture that have been attributed to the low implantation and pregnancy rates in earlier reports [39]. Furthermore, considering the replacement, reduction and refinement requirements of ethics in animal research, refinements in NSET devices have reduced the number of animals needed and the stress/pain they may experience during the procedure [49, 50].

We chose embryo transfer using the NSET device to reduce stress, recovery time and potential complications with anaesthesia and post-operative care. However, a further complication in this species is that *A*. *cahirinus* do not produce a visible seminal plug after mating [1, 19] and pseudopregnancy cannot be induced by traditional methods [25, 39, 51, 52]. Females therefore need to be subjected to daily vaginal lavage for 2–7 days and staged with H&E as reported previously [2]. Similar to humans [11], spiny mouse surrogates must be in the mid-late luteal phase of their menstrual cycle prior to ET [2], to provide the optimal uterine environment for embryo implantation. Because of significant species-specific differences that exist in reproduction between the mouse and spiny mouse, even well-defined laboratory techniques for the laboratory mouse require modification before they can be applied to *A*. *cahirinus*.

Considering the unique reproductive biology of *A*. *cahirinus* and its potential as a model for contraceptive discovery and human reproductive disorders, the development of successful protocols for assisted reproduction in spiny mice will provide a better understanding of its embryogenesis, implantation and pregnancy whilst acting as a more relevant small animal model for women’s reproductive health.

### Limitations and future directions

Being a comparatively new laboratory species, limited information is available regarding spiny mouse reproductive biology and physiology and we acknowledge several potential limitations to the use of the protocols we have outlined.

Data on changes in LH and FSH across the menstrual cycle in this species is currently unavailable because of the lack of specific LH and FSH immunoassays and, despite the use of Deslorin to suppress the oestrous cycle of various mammals including some rodents (reviewed by [53]) we are unable to determine in advance if Deslorelin will induce amenorrhea in the spiny mouse. Moreover, we are unable to collect standard, repeated blood-draws from ear or tail veins in this species due to the fragility of these tissues owing to the spiny mouse’s unique ability for skin autotomy [54]. Longitudinal studies of changes to ovarian steroid production within individuals are therefore currently not possible.

Similarly, while we have an understanding of follicular growth in the cycling spiny mouse [2], follicle dynamics following hormone treatment are unknown. Considering this, ovarian histology may prove useful in optimising administered hormone concentrations and provide insights into the potential role of the spiny mouse as a model for ovarian hyperstimulation syndrome (OHSS).

Spiny mice remain fertile until at least 2 years of age in our colony but a decline in fertility has been observed between 3 and 4 years of age (unpublished). While the ovarian hormone profile (estrogens and progesterone) of older females is unknown, the impact of exogenous hormonal stimulation in these animals is likely to be reduced if they are to experience similar changes during reproductive aging as other mammals [55–57]. Future Investigations of reproductive aging in this species may not only suggest a role in studies of human reproductive aging, but also in improving ovarian stimulation for women of advanced maternal age.

To our knowledge, embryo cryopreservation has not been attempted in this species and we cannot confirm that the cryoprotectants described in this protocol will successfully preserve spiny mouse embryos. If not successful, future studies may need to compare different concentrations of Ficoll, different permeating cryoprotectants or cooling rates on spiny mouse embryo cryopreservation to improve the success of embryo cryopreservation in spiny mice.

If successful, our protocols will benefit future in-vitro and in-vivo reproductive research in this species by providing viable cells and tissues to more effectively use the spiny mouse as a model for human reproduction.
